# A concrete future

**DOI:** 10.1038/s44172-022-00026-3

**Published:** 2022-11-03

## Abstract

Professor John Provis gives a perspective on cement and concrete technology, from ancient masterpieces to a vision of a sustainable future.

*John Provis* is a Professor of Cement Materials Science and Engineering at the University of Sheffield. His research focusses on the development, characterisation and exploitation of cement and concrete-based materials for construction, infrastructure and waste immobilisation applications. Here we ask Professor Provis for his perspective on these ubiquitous construction materials, from ancient masterpieces to a vision of a sustainable future.

**Figure:**
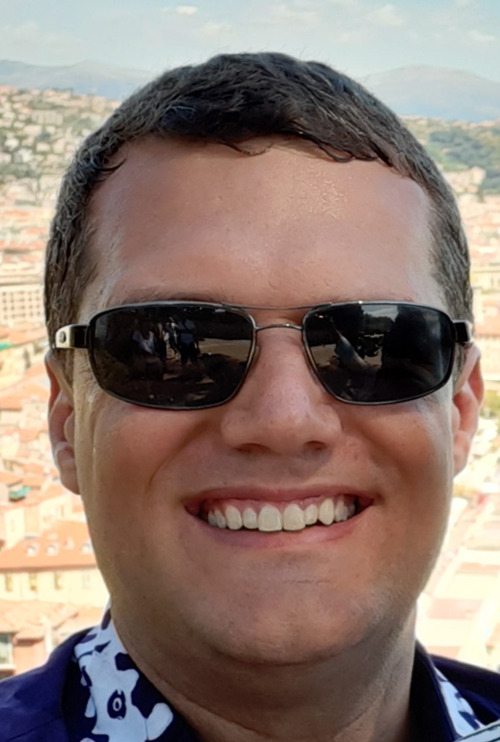
John Provis

What originally got you interested in cement science and technology?

I came into cements research almost by accident. I studied chemical engineering at the University of Melbourne, and was doing summer research work on a minerals processing project with Professor Jannie van Deventer. Jannie is a pioneer in the field of geopolymer cements and remains a good friend and mentor of mine. He persuaded me to do a final year project with him—and then midway through, he dropped a PhD offer on the table. It was an ambitious idea to develop a reaction kinetic model for how geopolymer cements react and harden. It immediately appealed to me because it was such a dive into the unknown. And the rest is (approximately) history!

What’s your favourite cement/concrete-based structure and why?

To me, the most impressive and spectacular concrete structure in the world is the Pantheon in Rome. It’s made with a stunning combination of architecture, engineering and materials science, including the choice of a lightweight concrete to build the dome on top. In many ways, it’s centuries ahead of its time, and has survived through 2000 years of earthquakes, wars and other calamities.

**Figure:**
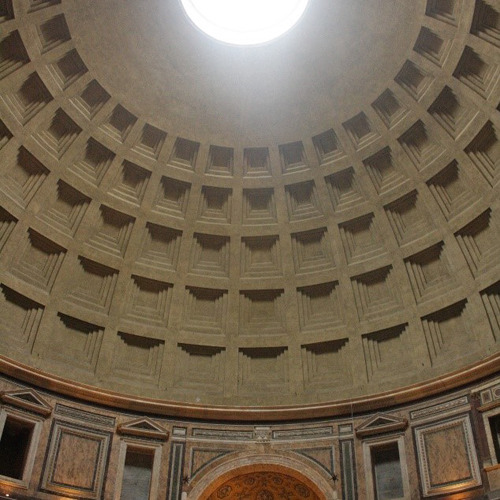
Pantheon dome, Rome. John Provis

What do you see as the biggest fundamental challenge/s in cement research at the present time?

The elephant in the room for the cement sector is sustainability. On average, everyone on the planet consumes (not physically) more cement than food. We use something like 1.5 kg of cement per  person per day. We can produce cement extremely efficiently, and in enormous volumes. However, the combination of these volumes plus the fact that we’re tied to limestone (calcium carbonate) as a raw material means that the CO_2_ footprint of cement production is several times greater than that of global aviation. As society very correctly places restrictions on highly emitting activities, we’re looking at some questions at an existential level regarding what we can or even should do with cements. There’s never going to be a like-for-like replacement for the conventional Portland cement that we’ve relied on since the 1800s for all of our construction needs, because there’s no way we can make the same volumes of any alternative material while being as inexpensive and as robust. This leads to the main theme of a large part of my research: moving the cements ecosystem toward a ‘toolkit’ of materials. Instead of aiming to replace Portland cement by a single more-sustainable option, my viewpoint is that we should develop a broad range of alternatives, depending on the raw materials that are available in each location, and the particular engineering and technical needs of the application. For example, why should we use the same cement to put in a fence post and to build a skyscraper? This is a huge change in philosophy for the construction sector. We can also make much better use of local raw materials, well demonstrated by the innovative work that is being done with local clay resources or volcanic ashes in many parts of the world. Or we can add value to local industrial wastes by using them to produce high-quality cements.

The other really fundamental challenge my research team works on is the safe and long-term immobilisation of nuclear wastes. In this context, we’re trying to design materials that need to do their job in protecting the biosphere and humanity for hundreds of thousands of years. This legacy is a strong motivation to get the science right. To build confidence in materials development for such practical applications we have to go to some deep and fundamental science, predicting material evolution over periods of millennia from thermodynamic principles, and building an understanding of how cements interact with wastes, groundwater, metallic canisters, and other materials they may encounter. These are not just future problems but very contemporary challenges with relevance for sites including Sellafield, Hanford, Fukushima, and others. This knowledge can also be carried over into sustainable construction, to motivate how we look at material design for durability in that context as well.

What do you see as key innovations/technologies/techniques that are transforming the field of cement and concrete research?

I think the key innovation is the realisation that we have to make and use cements and concretes more efficiently if we’re to get anywhere near meeting global emissions targets. This transformation in research is a move away from simply asking ‘how can I make stronger and more durable concrete?’ (which is a difficult question in itself), toward asking ‘how can I design a concrete that achieves its key performance targets with the minimum environmental cost’?

In this context we’re adding extra dimensions to each design or optimisation problem we look at. We’re replacing more and more of the conventional Portland cement in a concrete by other materials, most of which are wastes or by-products, and then modifying the chemistry with some cleverly designed additives and admixtures to get equal-or-better concrete performance in a much more efficient material. Getting this right requires high-precision nanoscience to understand surface interactions and mechanisms. We also need ever-improving characterisation techniques because cements are such a complex set of phases to identify and analyse. Finally, we need a good dose of engineering know-how to produce a concrete that’s usable on site. Concrete is one of the few high-demand, high-responsibility engineering materials that is, in most parts of the world, placed and finished by informally trained workers. This means that whatever we design at a scientific or engineering level has to be sufficiently robust and easy-to-use that it can be properly applied by the available workforce. This connection between the high-end science and the everyday practical application of one of the world’s most important and widely used materials has to run through everything we’re doing in construction materials research.

How is cement and concrete technology going to evolve? Any new applications?

I don’t envisage any major changes in cement and concrete applications as such; we’re still using concrete largely to do the same things as we did 100 years ago. Where I see the changes coming will be in processing routes: handling and placing concretes using digitally controlled processes (mixing, pumping, precasting under factory control, 3D printing or ‘smart’ casting in speciality applications, and so on) rather than the very manual and empirical procedures that are still used even for a lot of high-value and high-precision concreting work at present. I also expect that we will move much more toward using electrical energy rather than fossil fuels to produce cements. As part of this, we can store energy by converting it from electrical to chemical forms, by making either chemicals that will form part of the cement, or non-fossil fuels such as hydrogen to use in cement-making processes. This can then be linked with the decarbonisation of the electricity supply, to give an important pathway to reducing emissions across the cement and concrete sector.

In my view, materials research right now should provide solutions to society’s most pressing needs, rather than creating solutions (technologies or processes) that remain in search of a problem to solve. The provision of the infrastructure materials and processes for the future we want leads to many interesting research questions. There’s some incredibly cool science to be done along the way. But we can’t lose sight of the fact that the research we do now can and must sustainably serve global society for decades to come.

*This interview was conducted by Rosamund Daw, Chief Editor, Communications Engineering.*

